# Spatial–temporal evolution and correlation analysis between habitat quality and landscape patterns based on land use change in Shaanxi Province, China

**DOI:** 10.1002/ece3.10657

**Published:** 2023-11-01

**Authors:** Li Gu, Jiabo Yan, Yurong Li, Zhiwen Gong

**Affiliations:** ^1^ College of Forestry Northwest A&F University Yangling China; ^2^ College of Economics & Management Northwest A&F University Yangling China

**Keywords:** habitat quality, land use change, landscape pattern, spatial correlation

## Abstract

Regional habitat quality is an important reflection of ecosystem services and ecosystem health. Exploring the characteristics of habitat quality changes and revealing the vulnerability of regional ecosystems caused could provide reference for the improvement of ecological service functions and the protection of regional ecological environment. Based on remote sensing data of Shaanxi Province from 2000 to 2015, InVEST model and grid analysis were used to analyze the evolution characteristics of habitat quality and landscape pattern, and spatial autocorrelation was also used to analyze the spatial correlation and temporal evolution characteristics. The results showed: (1) Arable land, grassland, and forest land were the main landscape types in Shaanxi province, accounting for more than 94% of the total area, and the arable land and unused land showed a decreasing trend, while the grassland and forest land showed an increasing trend, and the proportion of construction land continued to increase with the rapid economic development from 2000 to 2015; (2) The spatial distribution characteristics of habitat quality was similar to land use cover change, which was “high in the southern and central forest areas, low in the northern sandy land and central urban agglomeration”, and habitat quality value showed a steady increase, indicating that the habitat quality was getting better; (3) The landscape pattern index values of Guanzhong Plain urban agglomeration changed significantly, which tended to be fragmented, and the landscape types were more diverse and uniform; (4) There were obvious spatial correlation between habitat quality and landscape pattern, and the spatial differentiation of clustering was obvious, and the clustering effect of habitat quality and landscape pattern characteristics would weaken with the increase in urbanization degree. The analysis of the spatial association between habitat quality and landscape pattern could provide scientific support for ecological protection and landscape planning.

## Graphical abstract

The habitat quality value in Shaanxi Province showed a steady increase, indicating that the habitat quality was getting better, and spatial distribution characteristics was “high in the southern and central forest areas, low in the northern sandy land and central urban agglomeration”, and there was obvious spatial correlation between habitat quality and landscape pattern.

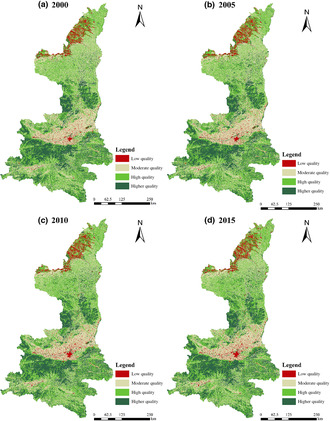

## 1 INTRODUCTION

Urbanization is a worldwide social and economic phenomenon accompanied by industrialization and economic development, and it is an important symbol and inevitable trend of human economic and social development (Ahmed et al., 2021). China has experienced the largest and fastest urbanization in the world (Li et al., 2020; Sun et al., 2021). However, with the acceleration of urbanization and industrialization, arable land, green space, water, and other land use types are continuously encroached, and the area of ecological patches is gradually reduced and the fragmentation degree is increasing (McDonald et al., 2009; Zhang et al., 2004). Some important corridors are also cut off and some key nodes disappear, which significantly changes the structure and function of the regional landscape (Chen et al., 2019). Ecological and environmental problems such as biological homogenization, ecosystem degradation, urban heat island effect, and air pollution occur frequently (Das et al., 2021), which seriously threaten regional ecological security and become a major bottleneck restricting urban sustainable development (Grumbine, 2004).

Landscape pattern is the concrete embodiment of landscape heterogeneity, and it is also the final result of various natural and human factors at different spatial and temporal scales (Dadashpoor et al., 2019; Mertz, 2016). The characteristics of landscape pattern change could affect the process of biodiversity and the distribution of ecosystem services (Maheng et al., 2021). Habitat quality is the ability of an ecosystem to provide suitable conditions for the sustainable survival of individuals and populations (Bakker et al., 2021; Li et al., 2022), and it is also an important index to reflect ecosystem service functions and evaluate regional ecological security. Habitat quality changes could visually show the changes of regional biodiversity and landscape pattern (Mortelliti et al., 2010), and the landscape spatial pattern could reflect the regional ecological environment quality (Gude et al., 2006). The two influences each other, and the corresponding changes of ecological environment quality could be obtained by studying the spatial and temporal changes of landscape pattern (Xiong et al., 2021).

The influence of climate change and human activities was the main reason for the large fluctuation of habitat quality (Peng & Wang, 2019). With the development of urbanization, landscape pattern/land use change, and habitat quality in many regions is generally declining (Gaylard et al., 2020). The changes of land use and habitat quality at different scales, basins, regions and cities, focusing on urban agglomerations, watersheds, coastlines, and hilly areas have been studied, and the impact of land use change on habitat quality was discussed, and the spatial–temporal evolution characteristics of habitat quality were analyzed by means of geographic detectors, buffer zones, and topographic gradients (Li et al., 2019; Wang et al., 2021). Analyzing the influence of construction land expansion on habitat quality could restore the ecological environment background and evolution law, improve the urban and rural planning quality and ecological control in metropolitan areas, and clarify the relationship between land use change and habitat quality degradation (Aneseyee et al., 2020). Especially, under the background of the urban green space degradation and global urban population density growth, the threat to the surrounding natural or semi‐natural habitat is increasing (Ahrends et al., 2015). Therefore, to explore the internal expansion and connectivity of landscape patches on the habitat quality is very important.

The recent research of habitat quality mostly focused on the spatial–temporal variation of habitat quality, the response of habitat quality change to land use change, and mainly focused on the rapid economic development areas or metropolitan areas, with little focus on the habitat evolution law and mechanism in the ecologically vulnerable areas in the central and western regions (Boykin et al., 2013; Huang et al., 2020; Zhu, Ding, et al., 2020; Zhu, Zhang, et al., 2020). Spatial autocorrelation analysis is one of the important methods in exploratory spatial analysis (O'Neill et al., 1999), which could be used to analyze the correlation between high and low values of habitat quality from the perspective of spatial–temporal evolution, and reveal the spatial correlation characteristics (He et al., 2017; Zheng & Li, 2022). However, the analysis of coupling landscape pattern and habitat quality change of urbanization process is not deep enough (Tripathy et al., 2021). In particular, when analyzing the correlation between landscape pattern and habitat quality change, most of them did not consider the spatial attributes of geographic phenomena, and the research results could not characterize the specific spatial scope of the significant correlation. Moreover, most of them failed to quantitatively study the evolution trend of the spatial correlation between habitat quality and landscape pattern from the time series. Therefore, it is necessary to analyze the correlation between landscape pattern change and habitat quality from the perspective of spatial and temporal evolution, so as to reveal the spatial correlation characteristics of landscape pattern change and habitat quality. In this paper, the evolution characteristics of habitat quality and landscape pattern in Shaanxi Province from 2000 to 2015 were analyzed based on the theories and methods of habitat quality assessment and landscape pattern analysis, and the spatial correlation between them was also analyzed by geostatistic statistical methods. The results of this study could provide scientific guidance for quantitative assessment of the impact of urbanization factors on habitat and detection of its spatial heterogeneity, and for formulating differentiated development strategies for urban land use planning and management, and realizing urban ecological security.

## 2 MATERIALS AND METHODS

### 2.1 Study area

**FIGURE 1:**
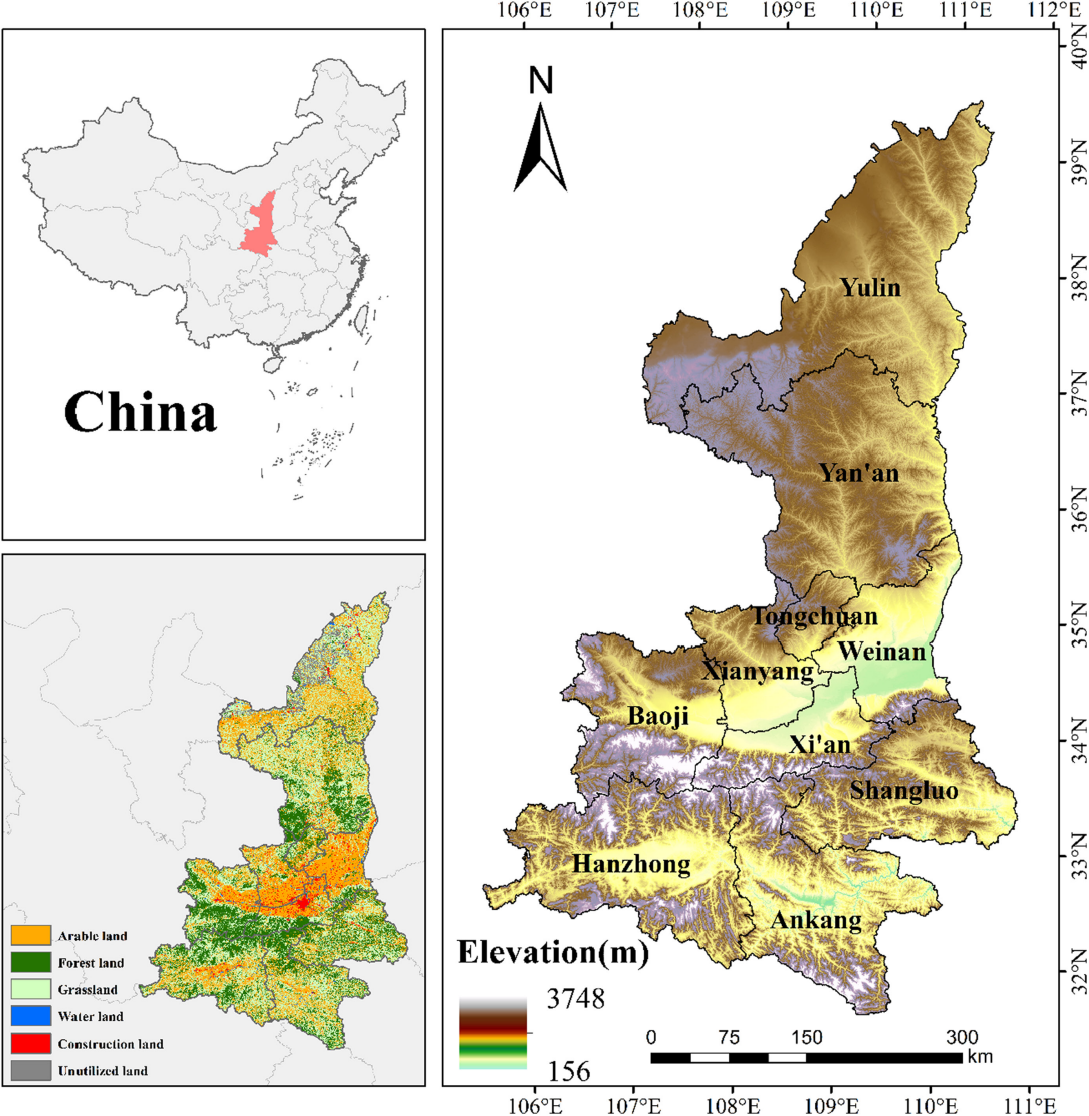
Location of the study area.

Shaanxi Province is one of the most economically vibrant and densely populated regions in Northwest China, but it is also with serious soil erosion and extremely fragile ecological environment, which is located in the middle reaches of the Yellow River, bordering Shanxi and Henan to the east, Ningxia and Gansu to the west, Sichuan, Chongqing, and Hubei to the south, and Inner Mongolia to the north. The total area of Shaanxi Province is about 20.56 × 10^4^ km^2^, and it is also the center of the world's largest and most typical Loess Plateau (Zhang et al., 2015) (Figure 1). The terrain is high in the north and south and low in the middle. It is composed of various land forms, such as plateau, mountain, plain, and basin, among which the Loess Plateau accounts for 40% of the total area, spanning the Yellow River and the Yangtze River and three climatic zones. There are 61 forest nature reserves, covering an area of 11.46 × 10^4^ km^2^, accounting for 5.6% of the total area of the province (Wang et al., 2013). There are also many ecological reserves, such as water and soil conservation, water conservation, wind and sand fixation, etc. It is also the main project area of the Three‐North Shelterbelt Program and the Yellow River Basin ecological protection project. The total population of the province was 39.54 million, and the total GDP was 2.980098 trillion yuan by the end of 2021 (Zhao et al., 2014). However, with the rapid economic development, the problems of local resources and environment have become increasingly prominent, and the ecological environment has become the focus of social attention.

### 2.2 Data source and processing

**FIGURE 2:**
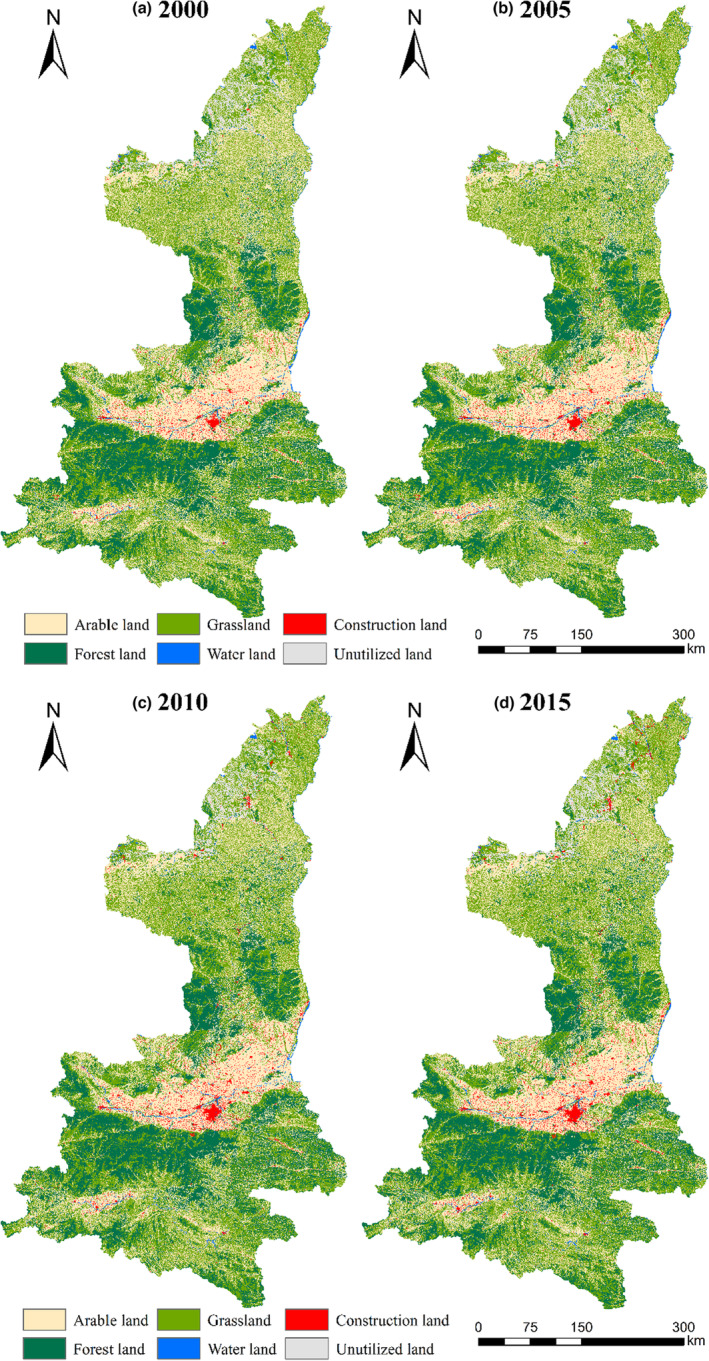
Spatiotemporal characteristics and variation patterns in Shaanxi Province from 2000 to 2015.

The data were obtained from the Resources and Environment Data Center of the Chinese Academy of Sciences (http://www.resdc.cn/), including land use data (in 2000, 2005, 2010, and 2015 with 30 m resolution), administrative boundary data, and DEM data (SRTM digital elevation data in GRID format with 30 m resolution). The generation of land use dataset is based on Landsat TM/ETM+ and OLS remote sensing images of each period, combined with human–computer interaction interpretation method, and verified in the field, the classification accuracy reaches more than 94.3% (Figure 2). According to the natural attributes and utilization attributes of land resources, the land use data can be divided into six first‐level land classes and 25 second‐level land classes (Appendix A: Table A1). According to the needs of habitat quality study, the marshes were classified as wetland/water body, and the other sandy land, Gobi, saline‐alkali land, bare land, and bare rock land were classified as unused land.

### 2.3 Analytical methods

#### 2.3.1 Habitat quality assessment of the InVEST model

The hypothesis of habitat quality model is that areas with higher habitat quality could support higher species richness, and a decrease in habitat quality would lead to a decrease in species diversity (Nicholas et al., 2011). The habitat quality calculation included four functions, the relative impact of each threat factor, the relative sensitivity of each land use/cover to each threat factor, the distance between land use/cover and the threat source, and the degree of legal protection of land. The formula is as follows (McGoff & Irvine, 2010):

(1) $$Q_{\mathrm{xj}}=H_{j}\left[1-\left(\frac{D_{\mathrm{xj}}^{z}}{D_{\mathrm{xj}}^{z}-k^{z}}\right)\right]$$

where $\;Q_{\mathrm{xj}}$ is the habitat quality index, the larger the value, the better the regional habitat quality and biodiversity service function; $H_{j}$ is the habitat suitability score of the landscape type; k is the coefficient of half‐saturation sum, first setting it to 0.5, and then running it a second time to set the value to 1/2 of the maximum degradation score; z is the proportion parameter reflecting spatial heterogeneity, and the general value is 2.5; $D_{\mathrm{xj}}$ is degree of habitat degradation, which is used to represent the degradation degree of a habitat due to the stress of threat factors. The formula is as follows:

(2) $$D_{\mathrm{xj}}=\sum_{r=1}^{R}\sum_{r=1}^{Y_{r}}\left(\frac{W_{r}}{\sum_{r=1}^{R}w_{r}}\right)r_{y}i_{\mathrm{rxy}}\beta_{x}S_{\mathrm{jr}}$$

(3) $$\begin{array}{c} i_{\mathrm{rxy}}=1-\left(\frac{d_{\mathrm{xy}}}{d_{r\max}}\right)\;\left(\text{The linear attenuation}\right) \end{array}$$

(4) $$\begin{array}{c} i_{\mathrm{rxy}}=\exp\left(\frac{-2.99d_{\mathrm{xy}}}{d_{r\max}}\right)\;\left(\text{Exponential decay}\right) \end{array}$$

where R is the number of threat factors to the habitat; y is the grid number of threat factors; $W_{r}$ is the weight; $Y_{r}$ is the total number of threat factor of *r* grid cells; $\beta_{x}$ is the reachability of x raster; $S_{\mathrm{jr}}$ is the sensitivity degree of *j* landscape to threat factor r; $d_{\mathrm{xy}}$ is the maximum threat distance of the threat factor; $i_{\mathrm{rxy}}$ is the influence distance of stress factor.

Landscape types with higher degree of human activities have greater impact on habitat (Deng et al., 2009). In order to make the related parameters including the threat factor data and habitat sensitivity combine with the actual situation of the research area as much as possible, the paddy field, dry land, urban land, rural residential areas and other construction land as habitat stress factor, and the maximum distance and weight of threat factors and the sensitivity of habitat to threat factors were set (Tables 1 and 2). The closer the habitat quality index (HQI) is to 1, the higher the habitat quality is, the more complete the regional habitat is, and the corresponding function and structure are, the more beneficial to the maintenance of biodiversity. In this paper, the habitat quality was divided into four sections using the equidistance method: low‐quality area (0 ≤ HQI < 0.3), medium‐quality area (0.3 ≤ HQI < 0.5), high‐quality area (0.5 ≤ HQI < 0.8), and higher quality area (0.8 ≤ HQI < 1.0).

**TABLE 1:** Threat factors and related coefficients.

The threat factors	Maximum stress distance/km	The weight	Type of spatial decay
Paddy fields	4	0.7	Exponential
Dry land	4	0.7	Exponential
Urban land	10	1	Exponential
Rural residential land	6	0.4	Exponential
Other construction land	8	0.8	Exponential
Unused land	3	0.2	Exponential

**TABLE 2:** Sensitivity of habitat types to each threat factor.

Land use types	Habitat suitability	The threat factors					
		Paddy fields	Dry land	Urban land	Rural residential land	Other construction land	Unused land
Arable land	0.4	0	0	1	0.5	0.7	0.5
Forest land	1	0.7	0.7	1	0.7	0.8	0.5
Grassland	0.8	0.4	0.4	0.7	0.5	0.6	0.8
Water land	1	0.6	0.6	0.9	0.7	0.7	0.4
Construction land	0	0	0	0	0	0	0
Unused land	0	0	0	0	0	0	0

#### 2.3.2 Landscape pattern index

Landscape pattern index is a simple quantitative index that could highly condense landscape pattern information and reflect the structural composition and spatial characteristics (Abdullah & Nakagoshi, 2007). In order to explore the interference degree of human activities on the landscape, combined with the ecological meaning of landscape index, five landscape pattern indices including largest patch index (LPI), edge density (ED), degree of separation index (DIVISION), Shannon diversity index (SHDI), and Shannon evenness index (SHEI) were selected (Solon, 2009; Zhang, Bi, et al., 2019; Zhang, Liu, et al., 2019; Zhang, Niu, et al., 2019; Zhang, Sun, & Shan, 2019).

(5) $$\mathrm{LPI}=\frac{\mathrm{Max}\left(a_{1}a_{2}\ldots \ldots a_{n}\right)}{A}$$

(6) $$\mathrm{ED}=\frac{\sum_{i=1}^{m}\sum_{j=1}^{m}P_{\mathrm{ij}}}{A}$$

(7) $$\text{DIVISION}=1-\sum_{i=1}^{N}\sum_{j=1}^{N}{\left(\frac{a_{\mathrm{ij}}}{A}\right)}^{2}$$

(8) $$\text{SHDI}=-\sum_{i=1}^{m}\left(p_{i}\ln p_{i}\right)$$

(9) $$\text{SHEI}=-\sum_{i=1}^{m}\left(p_{i}\times \log\left(2p_{i}\right)\right)$$

where *A* is the total landscape area; n represents the number of patches in a certain type of landscape; *N* represents the total number of landscape type patches; m is the number of types in the landscape; $a_{\mathrm{ij}}$ is the area of the $j_{\mathrm{th}}$ patch of class i. $P_{\mathrm{ij}}$ is the perimeter of the *j*_th_ patch of class i; $p_{i}$ is the ratio of patch type i occupied in the landscape.

#### 2.3.3 Spatial correlation analysis

Global spatial autocorrelation could reflect the correlation degree between a certain geographic phenomenon or certain attribute on a regional unit and the same phenomenon or attribute on a neighboring regional unit (Anselin, 1988), which is a degree measure of aggregation in a spatial domain (Gong et al., 2019; Yang & Chen, 2019). In this paper, 5 km × 5 km grid was used as the basic unit for zoning statistics of habitat quality and landscape pattern index. Based on GeoDA v1.20 software, the FID number of grids was used as the basic variable, and ROOK adjacency was selected to construct the spatial weight matrix (Yin et al., 2021). Then, bivariate global spatial autocorrelation is used to explore the spatial correlation characteristics of landscape pattern index and habitat quality partition statistics (Yang & Chen, 2019). The specific formula is as follows:

(10) $$\begin{array}{c} I=\frac{n\sum_{j=1}^{n}\sum_{j=1}^{n}w_{\mathrm{xj}}\left(x_{i}-\bar{x}\right)\left(x_{j}-\bar{x}\right)}{\sum_{j=1}^{n}\sum_{j=1}^{n}w_{\mathrm{xj}}\sum_{i=1}^{n}{\left(x_{i}-\bar{x}\right)}^{2}}=\frac{n\sum_{j=1}^{n}\sum_{j=1}^{n}w_{\mathrm{xj}}\left(x_{i}-\bar{x}\right)\left(x_{j}-\bar{x}\right)}{S^{2}\sum_{j=1}^{n}\sum_{j=1}^{n}w_{\mathrm{xj}}} \end{array}$$

(11) $$\begin{array}{c} S^{2}=\frac{1}{n}i=\frac{1}{n}{\left(x_{i}-\bar{x}\right)}^{2} \end{array}$$

(12) $$\bar{x}=\frac{1}{n}\sum_{i=1}^{n}x_{i}$$

where I is Moran index; n is the number of spatial units; *x* and $x_{j}$ are the observed values of region i and j respectively; $\bar{x}$ is the average value of spatial units; $W_{\mathrm{xj}}$ is the spatial adjacency relation of region i and j; $S^{2}$ denotes the variance of the observed values. The value of I is generally between [−1 and 1], and less than 0 indicates a negative correlation in space, while greater than 0 indicates a positive correlation in space. Zero means uncorrelated and randomly distributed. The closer the value is to 0, the weaker the global correlation between the two variables is (Anselin, 2002).

Local Moran index could be used to characterize the degree of correlation between the attribute values of a unit and neighboring units, and local indicators of spatial association (LISA) distribution map was drawn on the basis of *Z*‐test (Bongjoon & Taeyoung, 2016; Moran, 1950). In order to show the characteristics of spatial aggregation and differentiation of landscape pattern index and habitat quality, bivariate local spatial autocorrelation analysis was conducted on habitat quality and landscape pattern index. The formula is as follows:

(13) $$I_{i}=\frac{\left(x_{i}-\bar{x}\right)}{S^{2}}\sum_{j}W_{\mathrm{xj}}\left(x_{j}-\bar{x}\right)$$

where n is the number of spatial units; $x_{i}$ and $x_{j}$ represents the observed values of units i and j, respectively; $W_{\mathrm{xj}}$ is the spatial adjacency relation of regions i and j; $S^{2}$ denotes the variance of the observed values. In the process of processing, the average habitat quality value of each grid and the value of each landscape pattern index were still used as data sources, and bivariate LISA aggregation map was drawn on the basis of *Z*‐test (95% confidence), with each landscape pattern index as the first variable and habitat quality index as the second variable.

## 3 RESULTS

### 3.1 Overall characteristics of landscape types

Arable land, grassland, and forest land were the main landscape types in Shaanxi Province, accounting for more than 94% of the total area (Table 3). From 2000 to 2015, the grassland and forest land showed an overall increasing trend and went through three stages of “increase‐increase‐slightly decreasing”. The area of forest land increased to 23.38% in 2000, and then decreased slightly to 23.35% in 2015, which increased by 1623.09 km^2^ in recent 15 years. The proportion of grassland increased obviously at first and then decreased slightly, which were 37.83%, 38.00%, 38.37%, and 38.28%. Arable land and unused land decreased from 34.96% in 2000 to 33.12% in 2015. The proportion of landscape types in water areas and unused land was small, about 0.8% and 2.2%, respectively, and the overall range of change was small. The construction land increased continuously in recent 15 years, from 1.59% to 2.32%, which was related to the rapid economic development and the intensification of urbanization.

**TABLE 3:** Area of each land type in different years.

Land use types	2000		2005		2010		2015	
	Area/km^2^	P/%	Area/km^2^	P/%	Area/km^2^	P/%	Area/km^2^	P/%
Arable land	71,866.73	34.96	69,959.91	34.03	68,163.18	33.16	68,085.42	33.12
Forest land	46,381.14	22.56	47,543.04	23.13	48,054.86	23.38	48,004.23	23.35
Grassland	77,754.54	37.83	78,102.50	38.00	78,871.16	38.37	78,679.36	38.28
Water land	1636.81	0.80	1700.23	0.83	1642.76	0.80	1637.11	0.80
Construction land	3146.20	1.53	3510.27	1.71	4295.38	2.09	4583.49	2.23
Unused land	4770.09	2.32	4740.58	2.31	4529.27	2.20	4566.21	2.22

### 3.2 Landscape type transformation

**FIGURE 3:**
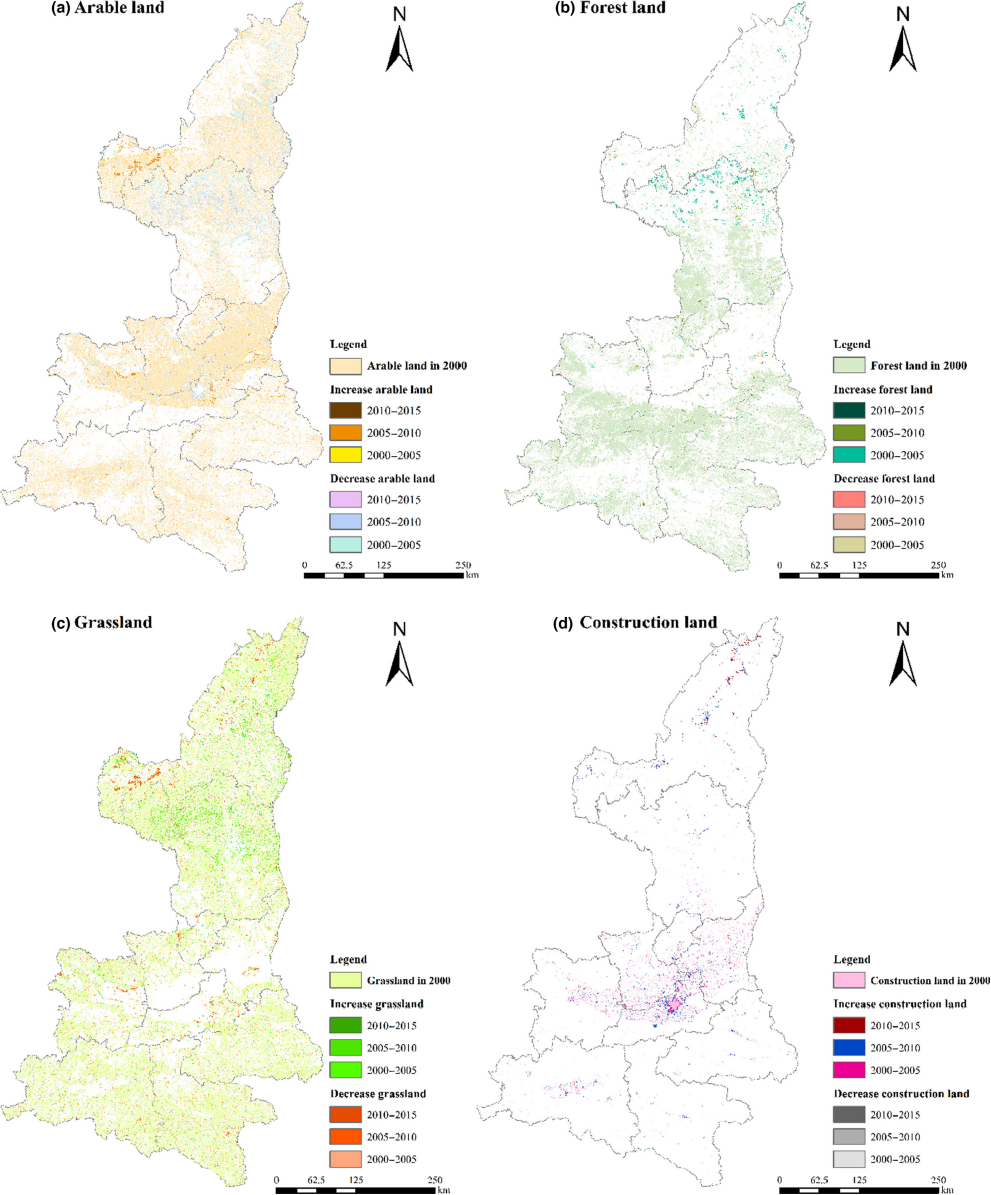
Spatial changes of major land use types during 2000–2015.

Land transformation changes mainly occurred in arable land, forest land, and grassland (Table 4). The transformation was mainly in the process of arable land to other land use types, among which the net transfer to forest land and grassland was 1001.32 and 1812.72 km^2^, respectively, which mainly occurred during 2000–2005. It is concentrated in the key areas and counties of “Grain for Green” Projects in northern Shaanxi (Figure 3a), with a net transfer of 1062.11 km^2^ to construction land and a final reduction of 3781.44 km^2^ of arable land. Forest land was mainly transferred from arable land and grassland, accounting for 56.58% and 41.51% of the total transferred amount, respectively, which mainly occurred during in 2000–2010 (Figure 3b). Grassland, was mainly converted to arable land, forest land, and construction land, accounting for 57.01%, 28.74%, and 7.52 of the reduced grassland area, respectively (Figure 3c). At the same time, all land types continue to transfer to construction land, resulting in a continuous increase in the area of construction land (Figure 3d). The net increase of 785.10 km^2^ from 2005 to 2015 is 2.07 times and 2.72 times of the other two periods, mainly from the transfer of arable land and grassland, concentrated in Guanzhong Plain urban agglomeration. It reflects the acceleration of regional urbanization; In summary, the general characteristics of land use change in Shaanxi Province from 2000 to 2015 are the transfer of arable land to forest land and grassland and the continuous increase in construction land.

**TABLE 4:** Land use change matrix from 2000 to 2015 (km^2^).

Year	Land use types	Arable land	Forest land	Grassland	Water land	Construction land	Unused land
2000–2005	Arable land	69,167.94	927.12	1362.98	71.78	331.86	5.03
	Forest land	96.80	46,102.64	149.63	4.22	14.81	13.03
	Grassland	626.46	505.23	76,513.34	27.32	25.88	56.27
	Water land	34.63	1.36	6.72	1593.16	0.63	0.31
	Construction land	8.99	0.91	3.03	0.64	3132.61	0.02
	Unused land	24.75	5.50	66.34	3.11	4.48	4665.92
2005–2010	Arable land	65,810.26	487.98	2847.18	92.81	704.42	17.25
	Forest land	288.15	46,915.93	261.02	8.58	50.54	18.83
	Grassland	1747.47	611.56	75,519.50	49.52	134.37	40.08
	Water land	129.27	9.81	76.65	1472.50	9.11	2.90
	Construction land	124.04	22.70	15.63	7.57	3340.29	0.05
	Unused land	63.96	6.87	151.16	11.78	56.64	4450.16
2010–2015	Arable land	67,169.33	123.97	612.01	31.58	203.33	22.76
	Forest land	140.04	47,659.29	221.70	3.81	12.15	17.56
	Grassland	643.95	212.71	77,793.00	14.51	100.29	106.43
	Water land	29.67	3.51	19.20	1584.40	4.88	1.10
	Construction land	45.94	1.84	6.15	0.87	4238.92	1.64
	Unused land	56.50	2.90	27.29	1.94	23.92	4416.72
2000–2015	Arable land	65,450.10	1316.99	3729.57	143.84	1192.31	33.83
	Forest land	315.67	45,676.40	263.38	12.26	71.84	41.49
	Grassland	1916.85	966.24	74,392.03	66.78	252.83	159.68
	Water land	141.23	10.25	75.93	1390.18	15.92	3.30
	Construction land	130.20	22.93	15.32	6.45	2971.15	0.16
	Unused land	131.16	11.35	202.82	17.58	79.43	4327.74

### 3.3 Variation characteristics of habitat quality

The mean values of habitat quality were 0.6759, 0.6795, 0.6812, 0.6800 from 2000 to 2015, which showed a steady increase and then a downward trend. The overall habitat quality was good, with high‐quality area and higher quality area as the main, and the total proportion of the two areas accounted for more than 61%. The proportion of medium habitat quality was also relatively large, and the proportion of low quality was the smallest. The area of low habitat quality continued to decrease from 34.96% in 2000 to 33.12% in 2015 (Table 5). The proportion of higher quality area increased first and then decreased. The high‐quality area continued to rise from 2000 to 2010, and then decreased slightly from 2010 to 2015, which may be related to the increase in construction. The proportion of high‐, higher, and low‐quality areas increased by 0.60%, 0.45%, and 0.78%, respectively, showing a two‐level differentiation state. From the perspective of the range of change, the range of change during 2005 to 2010 was the largest, and the area of low‐quality area, medium‐quality area, high‐quality area, and higher quality area respectively were 1.74, 23.19, 4.09, and 7.47 times of the area of change during 2010–2015.

**TABLE 5:** Area and ratio of each HQ level during 2000–2015.

HQ level	Value interval	2000		2005		2010		2015	
		Area/km^2^	P/%	Area/km^2^	P/%	Area/km^2^	P/%	Area/km^2^	P/%
Low	0–0.3	7892.37	3.84	8227.95	4.00	8799.65	4.28	9127.42	4.44
Moderate	0.3–0.5	71,866.73	34.96	69,959.91	34.03	68,163.18	33.16	68,085.42	33.12
High	0.5–0.8	77,615.25	37.76	77,961.32	37.93	78,742.55	38.31	78,551.36	38.21
Higher	0.8–1.0	48,181.15	23.44	49,407.36	24.04	49,851.23	24.25	49,791.61	24.22
Mean HQ		0.6759		0.6795		0.6812		0.6800	

**FIGURE 4:**
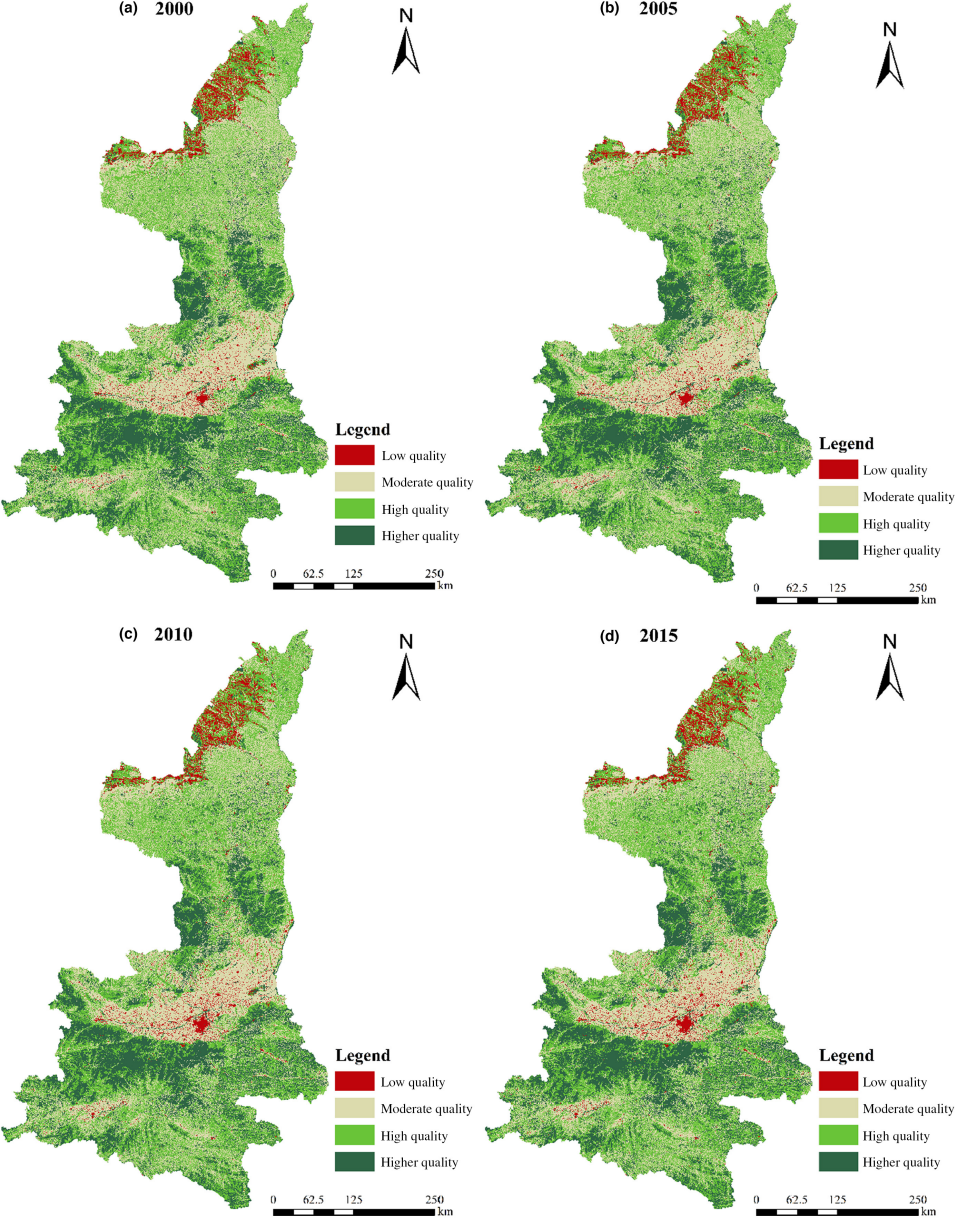
The changed of spatial distribution of habitat quality in Shaanxi Province from 2000 to 2015.

Habitat quality showed obvious spatial heterogeneity, with an overall distribution pattern of “high in the southern and central forest areas, low in the northern sandy land and central urban agglomeration” from 2000 to 2015, which was similar in LUCC (Figure 4). Higher quality area was mainly distributed in two areas, one is in the south of Yan'an, which is located in Huangling County, Huanglong County; the other is in southern Qinba Mountain region, which is located in Hanzhong, Ankang and Shangluo. In these areas, which were mainly for natural forest and national forest park, land use types were given priority to with forest land and grassland. It was not easy to be affected by threat sources such as arable land and various types of construction land, the degree of human disturbance was small, and the overall habitat quality was high. The high‐quality area and the medium‐quality area are mainly in the northern Shaanxi Plateau and in the central Guanzhong Plain, which were cross distributed, mainly the key districts and counties for the implementation of “Grain for Green” Projects around the urban agglomeration of Xi'an. The low quality was distributed in a large area in Yulin City, and matches with the distribution of construction land, especially urban land such as the main urban area of Xi'an City, Yan'an City, and Yulin City, both of them with high degree of human interference.

### 3.4 Evolution characteristics of landscape pattern

**FIGURE 5:**
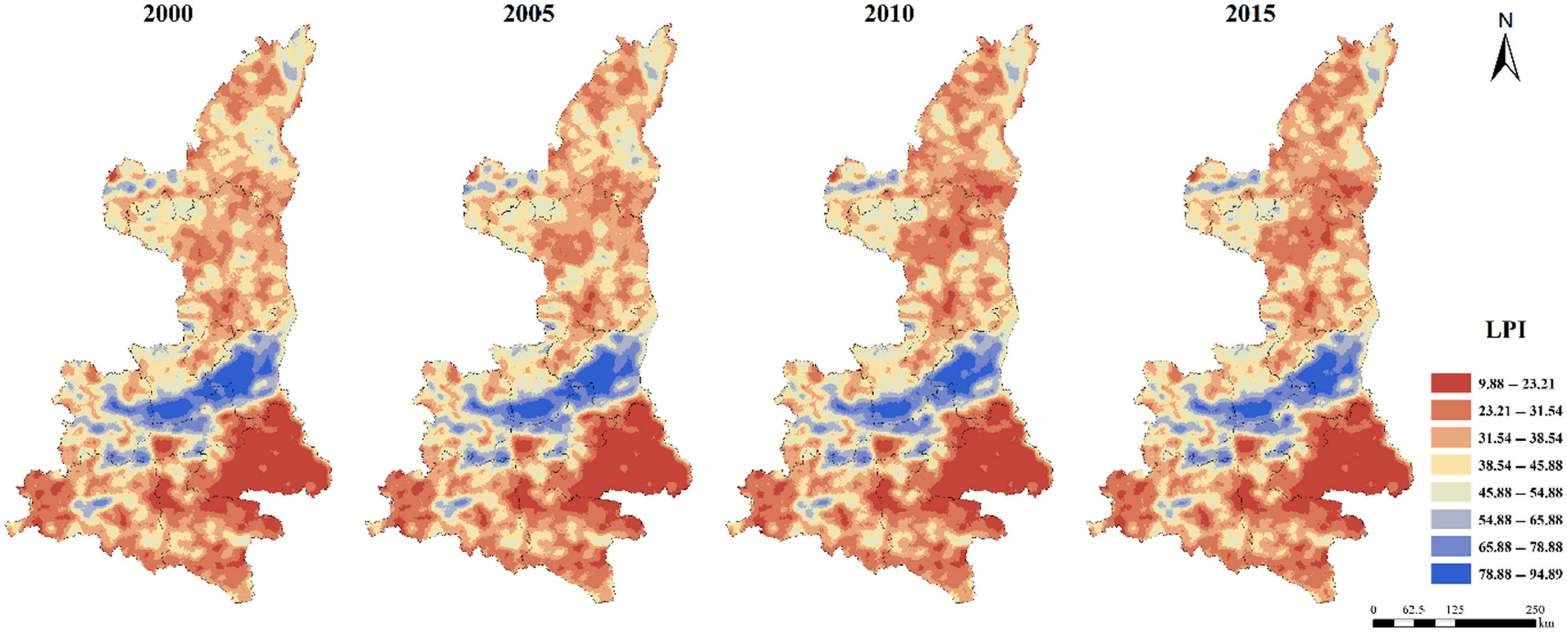
Spatial distribution of LPI index in Shaanxi Province from 2000 to 2015.

**FIGURE 6:**
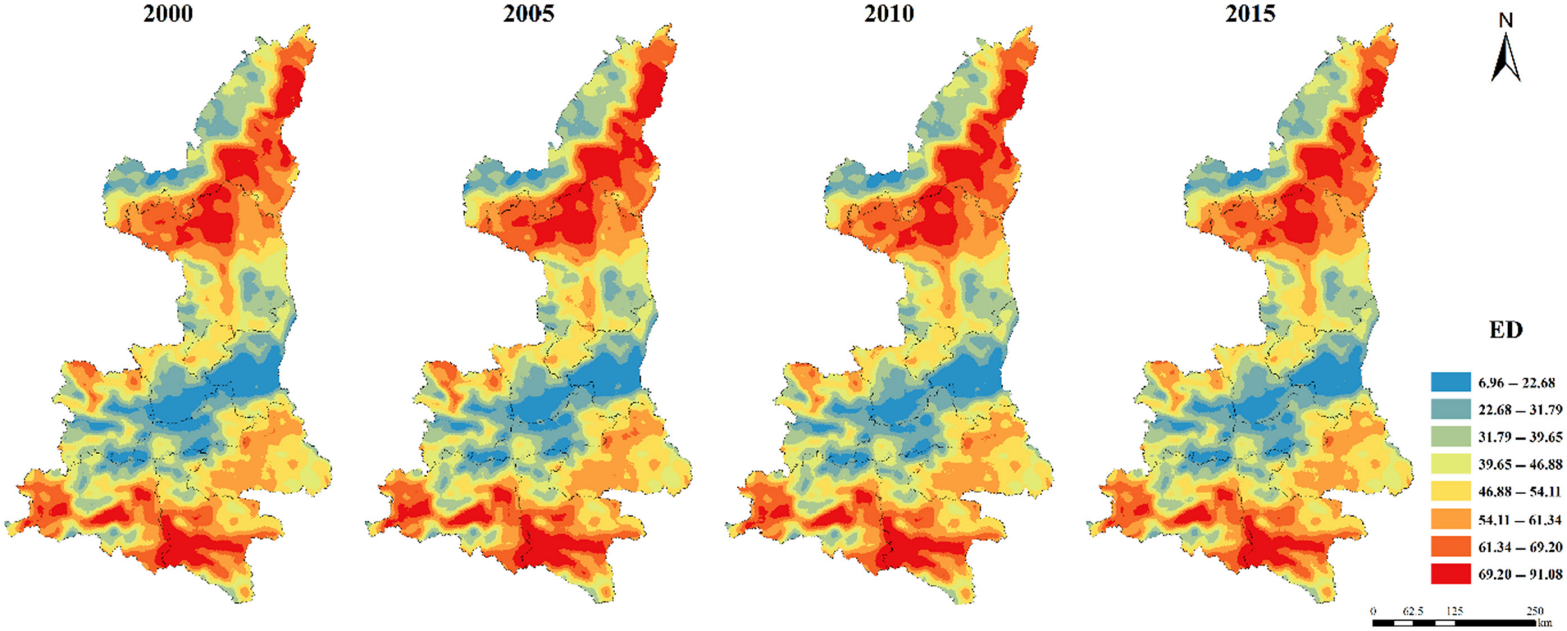
The changed of spatial distribution of ED index in Shaanxi Province from 2000 to 2015.

**FIGURE 7:**
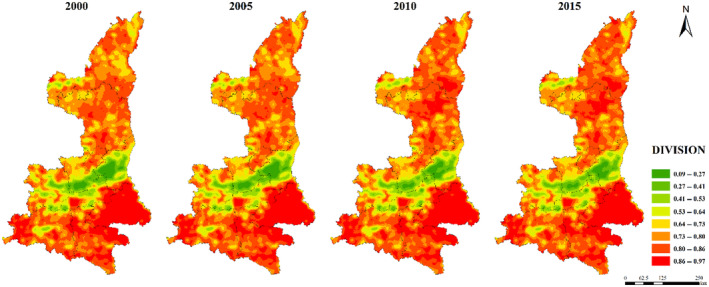
Spatial distribution of DIVISION index in Shaanxi Province from 2000 to 2015.

**FIGURE 8:**
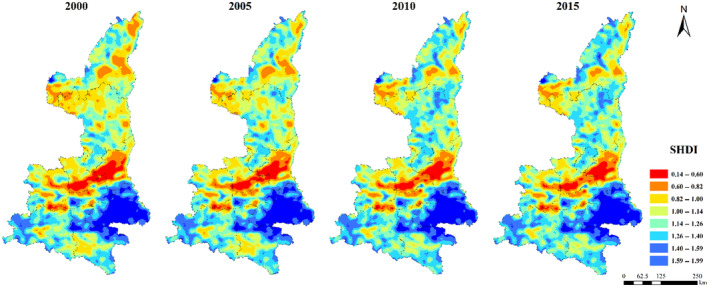
Spatial distribution of SHDI index in Shaanxi Province from 2000 to 2015.

**FIGURE 9:**
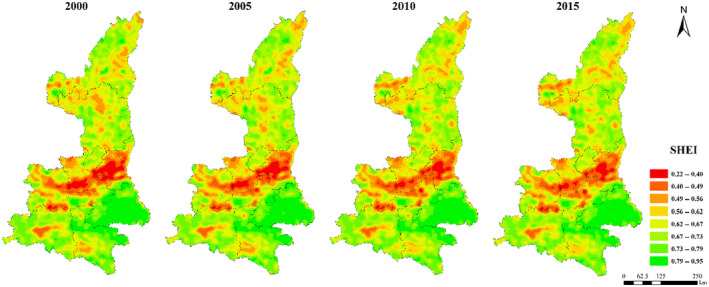
Spatial distribution of SHEI index in Shaanxi Province from 2000 to 2015.

The maximum patch area index (LPI) was generally low in northern Shaanxi Plateau and Qinba Mountain area, and high in Guanzhong Plain (Figure 5). The distribution of ED, DIVISION, SHDI, and SHEI were relatively consistent. The low values were concentrated in Guanzhong Plain urban agglomeration, while the high values were concentrated in Qinba Mountain area and the central Shaanxi Plateau (Figures 6, 7, 8, 9).

In the central region of northern Shaanxi, the edges of various patches have complex shapes, cross or network distribution between patches, and the dominant landscape patches have small area, diverse and evenly distributed landscape types, and high degree of landscape separation. In Northwest eolian sand grassland area, the landscape edge has complex shape, diverse types and uniform distribution, high degree of fragmentation, and high degree of separation of similar landscapes; In southern Qinba Mountain area and the central two mountain area (Ziwuling‐Huanglong Mountain), the edge shape of various patches is complex, the dominant patch area is small, and the landscape types are diverse and evenly distributed. This is because the natural forest conditions in mountainous areas restrict human activities. The landscape type is mainly natural landscape, while forest is the main landscape type. The landscape type is single and has no diversity. In urban agglomerations in Guanzhong plain, the advantage landscape patch area is large, edge smooth, landscape type singles no diversity, similar landscape in agglomeration effect, the area is located in the lower altitude, flat, as the main population, densely populated, Weihe Plain water is relatively abundant, human production activities with high intensity, degree is stronger for the modification of the natural landscape. Arable land is the main landscape type in this area, which has the characteristics of large scale and wide area. Therefore, the landscape patch area is large, the shape is regular and the landscape type is single. The high‐intensity land use pattern in urban areas also causes the dense distribution of various patches, which are divided by the road network, and the edge density is low.

### 3.5 Spatial correlation of landscape pattern and habitat quality

The landscape pattern index of LPI was negatively correlated with habitat quality (Table 6), while other indices were positively correlated, and all correlation analysis results were statistically significant (*p* < .01). The absolute value of correlation coefficients of each index showed a downward trend except for ED, which indicated that although there was a certain correlation between habitat quality and landscape pattern, the correlation between them was weakening.

**TABLE 6:** Correlation between landscape pattern index and habitat quality.

Year	Landscape index	Average	SD	Correlation	Significance
2000	LPI	39.18	20.22	−.309**	.000
	ED	47.85	17.46	.079**	.000
	DIVISON	0.76	0.18	.328**	.000
	SHDI	1.15	0.36	.376**	.000
	SHEI	0.67	0.16	.397**	.000
2005	LPI	38.81	20.10	−.305**	.000
	ED	48.81	17.89	.084**	.000
	DIVISION	0.76	0.18	.325**	.000
	SHDI	1.17	0.35	.363**	.000
	SHEI	0.67	0.15	.387**	.000
2010	LPI	38.27	20.02	−.285**	.000
	ED	48.52	17.56	.107**	.000
	DIVISION	0.77	0.18	.305**	.000
	SHDI	1.20	0.35	.336**	.000
	SHEI	0.66	0.15	.383**	.000
2015	LPI	38.21	20.02	−.277**	.000
	ED	47.99	17.15	.107**	.000
	DIVISION	0.77	0.18	.296**	.000
	SHDI	1.20	0.35	.318**	.000
	SHEI	0.66	0.15	.373**	.000

*Note*: ** indicates that landscape pattern index is significantly correlated with habitat quality at .01 level (two‐sided).

The spatial–temporal variation of habitat quality was significantly affected by land use change, and landscape pattern index and habitat quality have a certain correlation on the space (| Moran's *I* | between [1, +1], |*Z*| > 1.96), and the maximum patch area index (LPI) was negatively correlated with habitat quality in space, while the ED, DIVISON index, SHDI, and fragrance uniformity index (SHEI) were positively correlated with habitat quality in space (Table 7). From the perspective of Moran's *I* values, the spatial correlation between landscape pattern indices and habitat quality was relatively stable, and the change trend was consistent. In terms of time, except for ED, the other landscape pattern indices showed an obvious weakening trend, which was consistent with the above conclusion of bivariate analysis.

**TABLE 7:** Global Moran's *I* values for landscape pattern index and habitat quality in the study area from 2000 to 2015.

Year	Value type	LPI & HQI	ED & HQI	DIVISON & HQI	SHDI & HQI	SHEI & HQI
2000	Moran's *I*	−0.294	0.099	0.311	0.349	0.379
	*Z* value	−51.450	17.229	54.868	59.419	64.547
2005	Moran's *I*	−0.289	0.104	0.307	0.332	0.346
	*Z* value	−50.700	18.050	54.194	56.804	59.701
2010	Moran's *I*	−0.275	0.126	0.293	0.312	0.348
	*Z* value	−48.410	21.871	52.004	53.853	60.505
2015	Moran's *I*	−0.268	0.126	0.286	0.296	0.341
	*Z* value	−47.570	21.823	50.967	51.498	59.241

**FIGURE 10:**
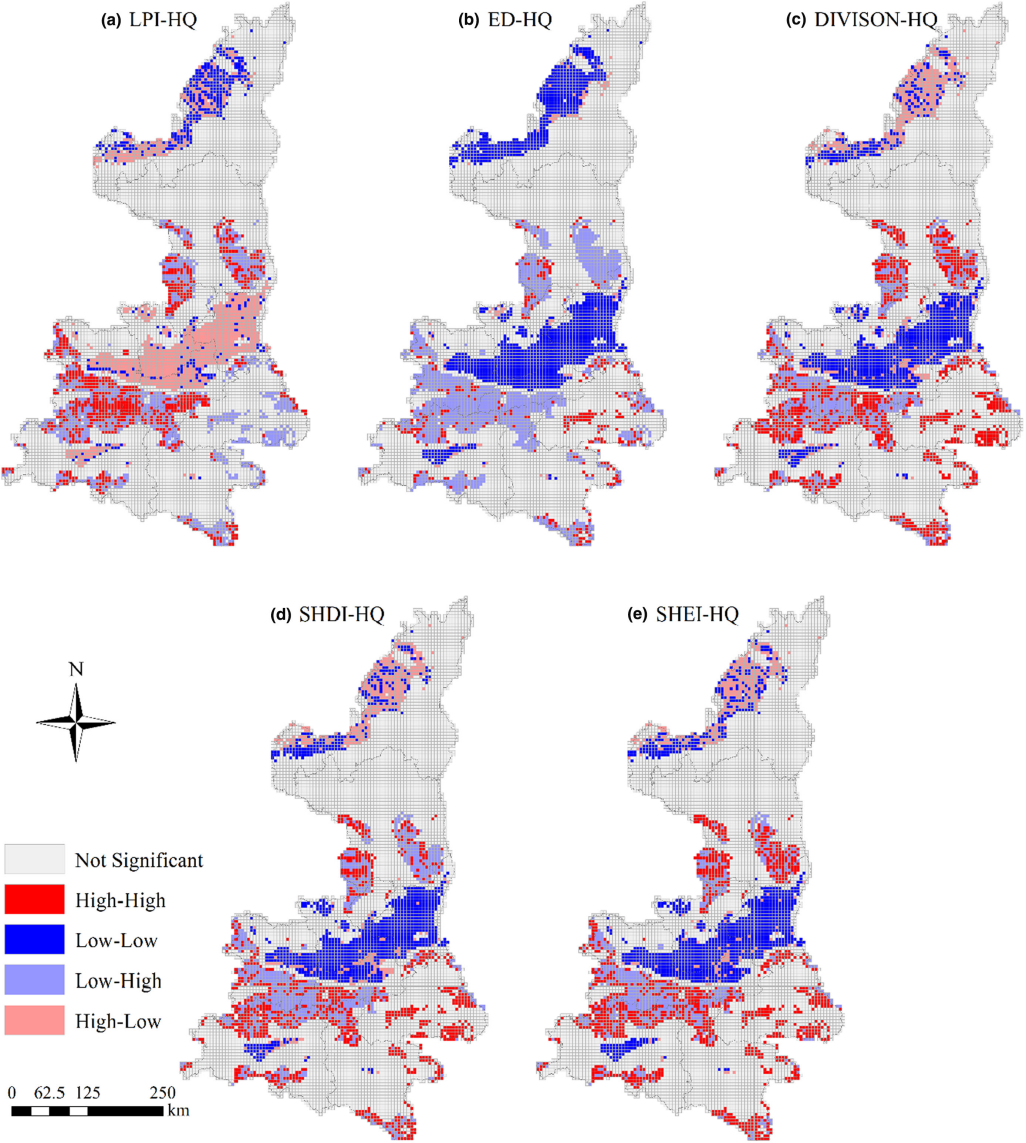
LISA cluster maps of landscape pattern index and habitat quality in Shaanxi Province.

LISA clustering map showed that there were five local spatial autocorrelation types: “high‐high”, “low‐low”, “high‐low”, “low‐high”, and “insignificant” (Figure 10). The H‐H type indicated that the high value of landscape pattern index and the high value of habitat quality had an agglomeration effect. The H‐L pattern indicated that the high value of landscape pattern index and the low value of habitat quality have agglomeration effect. For the LPI index, the H‐H type area was mainly distributed in the central and southern Qinling Mountains and the cross distribution of the L‐H type area. According to the LISA diagram of ED index, the L‐H type area mainly gathered in the central and mountainous areas and the Qinling Mountain area in the south, while the L‐L type area mainly distributes in the Guanzhong Plain urban agglomeration and the sandy area in the north. LISA plots of DIVISION, SHDI, and SHEI showed consistent distribution patterns, showing that the H‐H type clustered in the southern Qinling Mountain area and the Ziwuling‐Huanglong Mountain area in the central part, while the central Guanzhong Plain urban agglomeration was mainly the L‐L type.

## 4 DISCUSSION

### 4.1 Spatial pattern and evolution of habitat quality

Urban expansion causes isolation and fragmentation of landscape patterns, damages ecosystem integrity, and seriously affects the quality habitat (Balasooriya et al., 2009; Wang et al., 2020). Habitat quality in Shaanxi Province was consistent with the geomorphologic characteristics and spatial layout of land use types, and was also consistent with the existing research results, such in Sanjiang Plain area (Yang, 2021), Qilian Mountain area (Ren & Zhao, 2018), and Fujian Province (Liu et al., 2017). Due to the special geographic conditions of the Qinling national forest park and Qinba Mountain area, the disturbance degree of human beings is small, and all kinds of natural landscapes are good habitats for organisms, which were mainly in high quality habitats and in higher quality habitats (Zhang et al., 2021). In recent years, the Guanzhong Plain has witnessed rapid economic development and rapid urban expansion (Ye et al., 2022). The invasion of various artificial landscapes has seriously threatened the surrounding habitats, resulting in the low‐quality habitats.

Different from other studies on the continuous decrease of habitat quality with the rapid advancement of urbanization (Morel‐Journel et al., 2018), the overall trend of habitat quality in Shaanxi Province was first rising and then stable. On the one hand, the higher and high habitat quality areas increased, which was consistent with the increase in forest land and grassland and the decrease in arable land. Forest land and grassland are the main land types that provide quality habitat, and increasing the area of forest and grassland could promote the improvement of quality habitat. The reduction of arable land as a threat source reduced the threat to other habitats and improved the quality of local habitats. Therefore, major ecological restoration projects such as “Grain for Green” Projects, and ecological protection of Qinling Mountains and Yellow River basins were implemented (Zhang et al., 2018). As a result, the area of arable land, sandy land, and other land types as habitat threat factors decreased, and forest land and grassland as the main land types of high quality habitats increased, which improved the habitat quality and played a positive role. On the other hand, the area of low‐quality area continued to increase. The rapid development of human society and economy and the rapid advancement of urbanization process led to the continuous increase in construction land, and some high quality habitat patches gradually transformed into construction land patches as habitat stress factors (Song et al., 2020). The negative impact continued to intensify with the advance of time, further leading to the continuous decline of habitat quality.

However, the positive effect was greater than the negative effect, which made the habitat quality rise first and then steady (Yang, 2021). Therefore, the improvement of regional habitat quality should be done from two aspects in the future. One is to reduce the increase in major threat factors. Rapid urban expansion and agricultural development are dominated by the occupation of arable land, wetlands, and water bodies. As more such lands are included in the ecological red line and permanent capital arable land protection, agricultural expansion would shift to lands with weaker ecological functions, such as saline‐alkali land (Wang et al., 2019). This development may increase the amount of arable land in the area, but at the same time improve the soil environment and regulate salt content, thus providing better habitats for animals and plants. Second, ecological protection and restoration measures should be further strengthened (Zhang, Bi, et al., 2019; Zhang, Liu, et al., 2019; Zhang, Niu, et al., 2019; Zhang, Sun, & Shan, 2019). The urban development boundary could be demarcated to control the expansion of construction land and effectively control the new construction land. In addition, the use of pesticides and fertilizers should be reduced in the arable land concentration areas to effectively control agricultural nonpoint source pollution so as to improve the ecological quality with low habitat value (Liu et al., 2021).

### 4.2 Correlation between habitat quality and landscape pattern

Land use change led by human activities is one of the important factors leading to habitat quality change (Deng et al., 2009). The rapid urbanization process leads to drastic changes in the mode, intensity, and pattern of the original land use, which destroys the quality of the original habitat, reduces the landscape connectivity, and intensifies the land fragmentation, causing a huge impact on the structure, function, service capacity, and health status of the regional ecosystem (Solon, 2009). Spatial correlation between habitat quality and landscape pattern characteristics gradually weakened, and the relationship between the landscape pattern index and habitat quality is mainly affected by the dominance of landscape type (Zhu, Ding, et al., 2020; Zhu, Zhang, et al., 2020). The main reason was that the main low‐value cluster areas of habitat quality were distributed in the Guanzhong Plain urban agglomeration, which was the core area of rapid urbanization, and the land use types became more diversified, which complicated the shape of landscape elements such as large area of flat arable land and construction land, increased the landscape types, and improved the evenness (Fu et al., 2022).

Clarifying the impact of urban expansion on regional habitat quality has become an important theoretical basis for maintaining regional ecological security and ensuring high quality urban development (Zhu, Ding, et al., 2020; Zhu, Zhang, et al., 2020). The rapid expansion of satellite cities and county towns in metropolitan areas has assumed some functions of core urban land use. Similar artificial landscape elements have also begun to spread to the surrounding areas from a high concentration, so the degree of landscape separation and heterogeneity has gradually recovered (Mertz, 2016). In other words, with increased urbanization degree, the landscape pattern index seems to gradually approach the direction of natural landscape pattern index. However, even if the landscape patter changes, the diversified artificial landscape will still threaten the surrounding habitats. The habitat quality will not “improve” synchronously with the landscape pattern index (Yang, 2021). Therefore, the spatial correlation will decrease at any time in highly urbanized areas. Overall, the relationship between habitat quality and landscape pattern have certain regional differences, for advantage in natural landscape areas, the natural landscape diversity, complex shape features have a promoting effect on the habitat quality (Zheng & Li, 2022), and in the artificial landscape advantage for landscape area, habitat quality and has a certain relationship of landscape fragmentation. However, the spatial correlation between the two will weaken at any time.

## 5 CONCLUSIONS

The distribution of habitat quality had a high matching degree with the spatial distribution of topography and geomorphology, which showed the overall spatial distribution characteristics of “high in the southern and central forest areas, low in the northern sandy land and central urban agglomeration”. Landscape pattern was relatively stable, the landscape pattern of the mountainous area did not change significantly, and the landscape pattern index values of the Guanzhong Plain town group changed significantly. The diversity of artificial landscape makes the boundary of the Loess Plateau and Guanzhong Plain complex, the degree of separation of similar landscapes is increased, the landscape tends to be fragmented, and the landscape types are more diverse and uniform; there was an obvious spatial correlation between habitat quality and landscape pattern characteristics. In areas with natural landscape as the dominant landscape, landscape types, complex shape, uniform distribution, and other characteristics had promoting effects on habitat quality, while in areas with artificial landscape as the dominant landscape, the clustering effect of habitat quality and landscape pattern characteristics would weaken with the increase in urbanization degree.

## AUTHOR CONTRIBUTIONS

**Li Gu:** Writing – original draft (lead). **Jiabo Yan:** Formal analysis (supporting); software (equal). **Yurong Li:** Data curation (lead). **Zhiwen Gong:** Writing – review and editing (equal).

## CONFLICT OF INTEREST STATEMENT

The authors declare no competing financial interests.

## Data Availability

The data were obtained from the Resources and Environment Data Center of the Chinese Academy of Sciences (http://www.resdc.cn/).

## APPENDIX A

**TABLE A1:** Content and meaning of land use classification.

First‐level type		Second‐level type		
Number	Name	Number	Name	Meaning
1	Arable land			Refers to land on which crops are grown, including mature arable land, newly opened land, and recreational land, rotational land, grassland rotational crop land; land mainly planted with crops for agricultural fruit, agricultural mulberry, agriculture and forestry; beach land and sea shoals cultivated for more than 3 years
		11	Paddy field	Refers to arable land with guaranteed water sources and irrigation facilities, which can be irrigated normally in normal years, and which is used to grow rice, lotus root, and other aquatic crops. Cultivated land for growing aquatic crops such as rice, lotus root, etc., including the cultivated land where rice and dryland crops are rotated. This includes arable land where rice and dryland crops are rotated
		12	Dry land	Refers to cultivated land with natural water growth without irrigation water source and facilities; cultivated land for normal irrigation with water source and watering facilities; cultivated land mainly growing vegetables; recreational land and resting land
2	Forest land			Refers to forestry land where trees, shrubs, bamboo, and coastal mangroves grow
		21	Forest land	Refers to natural forests and plantations forests with crown density over 30%. Including timber forest, economic forest, shelter forest, and other patches of woodland
		22	Shrubs	Refers to the dwarf forest land and shrub woodland with crown density over 40% and less than 2 m in height
		23	Sparse wood land	Refers to the forest land with 10%–30% crown density of forest
		23	Other woodland	Refers to the young afforested land, cut‐over land, nursery and all kinds of garden land
3	Grassland			Refers to all kinds of grassland with more than 5% coverage, including shrub grassland mainly grazing and sparse forest grassland with less than 10% crown density
		31	High‐coverage grassland	Refers to coverage over 50% of natural grassland, improved grassland and mowing fields. This kind of grassland generally has better water conditions, and the grass is grown densely
		32	Medium‐coverage grassland	Refers to the natural grassland and improved grassland with a coverage level of over 20%–50%, which generally lacks water and has sparse grass
		33	Low‐coverage grassland	Refers to the natural grassland with a coverage of 5%–20%. This kind of grass lacks water, grass is sparse and animal husbandry utilization conditions are poor
4	Water land			Refers to natural land waters and land used for water conservancy facilities
		41	River and channel	Refers to the naturally formed or artificially excavated river and the main land below the perennial water level
		42	Lakes	Refers to the naturally formed water‐logged area below the perennial water level of the land
		43	Reservoir pit pond	Refers to the artificially built water storage area below the perennial water level of the land
		44	Permanent glaciers and snow	Refers to the land perennial covered by glaciers and snow
		45	Tideland	Refers to the tidal flooding zone between the high and low tide levels of high tides along the coast
		46	Mudlat	Refers to the land between the level of flat waters and the level of flood waters of rivers and lakes
5	Construction land			Refers to the urban and rural residential areas and other industrial, mining, transportation and other lands
		51	Urban land	Refers to the land used in large, medium, and small cities and county and towns built‐up areas
		52	Rural residential areas	Refers to rural settlements independent of cities and towns
		53	Other construction land for use	Refers to factories, mines, large industrial zones, oil fields, salt fields, quarries, as well as traffic roads, airports, and special land for use
6	Unused land			Land that is currently unused, including land that is difficult to use
		61	Sand	Refers to the land covered by sand with vegetation coverage below 5%, including deserts, excluding deserts in the water system
		62	Gobi	Refers to the land where the ground is mainly broken gravel and the vegetation coverage is less than 5%
		63	Saline‐alkali Soil	Refers to the land where the surface salinity accumulates and vegetation is scarce and can only grow strong salinity tolerant plants
		64	Marsh land	Refers to the flat and low‐lying terrain, poor drainage, long‐term moisture, seasonally ponded or perennial ponded and wet plants growth on the surface
		65	Naked land	Refers to the land covered with surface soil and vegetation coverage of less than 5%
		66	Naked rock texture	Refers to the land where the surface is rock or gravel and its coverage is more than 5%
		67	Else	Refers to other unused land, including alpine desert, tundra, etc.
